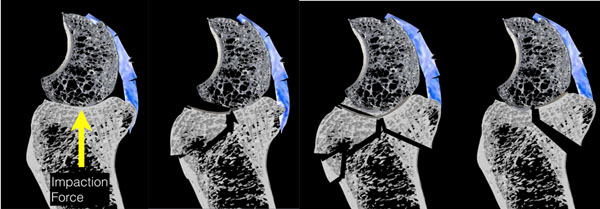# Abraham Colles oration: 200 years on

**DOI:** 10.1186/1753-6561-9-S3-A33

**Published:** 2015-05-19

**Authors:** Greg Bain

**Affiliations:** 1 Department of Orthopaedic Surgery, Flinders University of South Australia, 5042, Australia

## 

It is 200 years since Abraham Colles published his famous article where he described the common dorsal deformity of the distal radius fracture with an associated dislocation of the distal radio-ulnar joint. He provided a detailed description of the fracture, 80 years before the developed of radiographs. The fracture still bares his name, despite the fact that this article was never cited during his lifetime.

He described a method of reduction and application of the tin and wooden splint, to provide a good outcome. Failure to do so will lead to “ many months, of considerable lameness and stiffness of the limb, accompanied by severe pains on attempting to bend the hand and fingers.” The consolation is that “at some remote period the patient will again enjoy perfect freedom in all its motions, completely exempt from pain, however the deformity will remain undiminished through life”

The presentation will be directed to the anatomical aspects of the distal radius and how it fractures. This will include the gross and micro-architecture of the distal radius.

**Figure 1:**
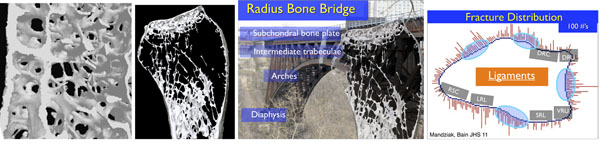


The concept of Osteo-ligamentous fragments will be discussed. The importance of the ligamentous attachments in initiating and propagation of fractures and their involvement in the instability patterns. Understanding these processes assists in planning the reduction and fixation of fractures of the distal radius, and hopefully prevents the many months, of considerable lameness and stiffness of the limb that otherwise can occur.

**Figure 2:**